# Evaluation of Alveolar Bone Destruction Patterns in the Posterior Region of the Maxilla Through Cone Beam Computer Tomography on 361 Consecutive Patients: Effect of Age and Gender

**DOI:** 10.1002/cre2.70000

**Published:** 2024-11-13

**Authors:** Filiz Namdar Pekiner, Gözde Yılmaz, Gaye Keser, Tan Fırat Eyüboğlu, Mutlu Özcan

**Affiliations:** ^1^ Department of Oral and Maxillofacial Radiology, Faculty of Dentistry Marmara University Istanbul Türkiye; ^2^ Private Practice; ^3^ Department of Endodontics, Faculty of Dentistry Istanbul Medipol University Istanbul Türkiye; ^4^ Clinic of Masticatory Disorders and Dental Biomaterials, Center of Dental Medicine University of Zurich Zurich Switzerland

**Keywords:** alveolar bone, combined periodontal–endodontic lesion, cone beam computed tomography, diagnostics, maxillary bone, periodontal defect

## Abstract

**Objectives:**

The aim of this retrospective study is to evaluate the effect of age and gender on the alveolar bone destruction pattern with cone beam computed tomography (CBCT) in the posterior region of the maxilla.

**Materials and Methods:**

The study group included CBCT image records of 361 consecutive patients (180 males and 181 females) aged 20 years and older. Alveolar crest morphology in the maxillary right and left first and second molar teeth on retrospective images was classified as a horizontal or vertical defect (one‐walled, two‐walled, three‐walled, and combined bone defect) on four surfaces (mesial, distal, buccal, and palatinal). Bone crater defects were defined, and furcation involvements and combined periodontal–endodontic lesions (CPELs) were placed in another category.

**Results:**

In 361 patients, 1444 teeth were evaluated from adults between 20 and 63 years of age; 49.9% of the patients were male and 50.1% were female. Female patients had a considerably greater rate of one‐walled horizontal damage in the right molar teeth than male patients (*p* = 0.002; *p* < 0.05). Patients with combined horizontal destruction in the right and left molar teeth, horizontal destruction in the palatinal, and horizontal three‐walled destruction had a significantly higher mean age than patients without these periodontal destructions (*p* = 0.000; *p* < 0.05). Males were shown to have statistically higher frequencies of horizontal defects when defects were combined or distally and palatally located.

**Conclusions:**

Age and gender affect the alveolar bone loss pattern. Except for single‐walled destructions, it has been found that the frequency of horizontal destruction increases with age. Horizontal destruction in the palatinal along with horizontal three‐walled destruction increased with age.

## Clinical Relevance

Recognizing the alveolar bone destruction patterns and affecting demographic factors in the maxillary molar area, the most susceptible region to periodontal abnormalities, may provide a better understanding of alveolar bone loss with accurate diagnosis and effective treatment planning.

## 1 Introduction

Periodontitis is characterized by microbially associated, host‐mediated inflammation that results in loss of periodontal attachment (Caton et al. 2018). Although periodontitis is an infectious illness of the gum tissues, the alteration that it causes in the alveolar bone is particularly of concern because of tooth loss caused by alveolar bone degeneration. Advanced periodontal disease is characterized by alveolar bone loss (Newman et al. 2012; Bagis et al. 2015).

A precise assessment of bone morphology is required for periodontal disease diagnosis, treatment planning, and prognosis (Langen et al. 1995). Detailed examination in the region cannot be established using intraoral or extraoral two‐dimensional imaging techniques used in periodontology and diagnostics due to the superposition of anatomical features. This scenario makes it difficult to diagnose periodontal disease and prevents accurate assessment of the alveolar bone level. Inability to detect vertical bone defects is a concern in this respect. As a result, it is essential to obtain thorough information using three‐dimensional imaging techniques (Bagis et al. 2015; Farman 2007; Saberi et al. 2017; Şener and Baksı 2013).

With advancements in medical technology, low‐dosage computed tomography (CT), namely, cone beam CT (CBCT), has increasingly been used in dental practice, particularly since the 2000s (Orhan 2012). CBCT is utilized in numerous fields, including orthodontics and periodontology, to assess the size and volume of bone lesions and to plan graft treatments in these regions (Orhan 2012; Scarfe, Farman, and Sukovic 2006; Hassan and Jacobs 2008). The superiority of CBCT compared to intraoral radiographs was reported in previous studies in the detection of periodontal abnormalities (Bayat, Talaeipour, and Sarlati 2016; Zhang, Rajani, and Wang 2018). Hence, the use of CBCT to observe the alveolar bone loss on one of the most affected alveolar structures, the molar maxillary area, may provide vital information to better understand the extent of alveolar loss and the factors affecting this.

The effect of age and gender on alveolar bone loss may play a critical role in different periodontal diseases, as previously reported using periapical radiographs (Hou et al. 2002; Yusof 1990). However, the use of CBCT as a primary focus on the effect of age and gender on alveolar bone loss may provide a new perspective on the topic. Therefore, the purpose of this retrospective study is to compare the alveolar bone breakdown pattern in the posterior maxilla with CBCT images on the basis of age and gender.

## 2 Materials and Methods

This study was carried out according to the recommendations of the Declaration of Helsinki, and the study protocol, numbered as 09.2018.136, was approved by the Clinical Research Ethics Committee, Marmara University Faculty of Medicine, on February 2, 2018. The study group included CBCT (Planmeca Promax 3D Mid, voxel size of 0.4 mm) images available in the archive of 361 patients, 181 women, and 180 men, over 20 years old, who presented to Marmara University Faculty of Dentistry, Department of Oral Diagnosis and Radiology, between 2018 and 2020. Informed consent was obtained from all subjects and/or their legal guardian(s) for use of their data for research purpose.

In our study, 1000 panoramic radiographs were retrospectively evaluated on the basis of the specified criteria, and the available retrospective CBCT images of patients who met the study criteria were analyzed according to the classification parameters. According to the evaluation of panoramic radiographs, the study group included individuals with maxillary right and left molars, with a 2 mm margin between the enamel–cementum border and the alveolar crest, and without a cyst, tumor pathological lesion, developmental anomaly, or trauma history in this region. This study examined 1444 teeth (722 right and 722 left) of 361 patients.

### 2.1 Radiological Interpretation

In the present study, limited fields of view of 8 cm × 8 cm, 8 cm × 16 cm, and 12 cm × 8 cm were used for all CBCT scans, and the data were reconstructed with slices arranged 0.25 mm apart. The MSI NB GL62M 7RDX‐1837XTR 17‐7700HQ 8GB DDR4 GTX1050 GDDR5 2GB 1TB15,6 FHD DOS 1920 × 1080 pixels, was used in a darkroom to evaluate the CBCT maxillary scans. To achieve the maximum visualization, the software's image processing function was used to improve the images' contrast and brightness. All patients received the same exposure and positional instructions.

CBCT images were used to classify alveolar crest morphology in maxillary molar teeth as mesial, distal, buccal, and palatinal on four surfaces, as well as horizontal and vertical defects as one‐walled, two‐walled, three‐walled, and combined defects. Bone crater defects on the buccal and palatinal surfaces of the teeth were characterized, and dehiscence and fenestration in the alveolar bone were examined in the interdental area. Furthermore, combined periodontal–endodontic lesions (CPELs) and Grade 1, 2, and 3 furcation involvements were assigned to a separate group.

Periodontal disease begins with gingival inflammation and progresses to alveolar bone deterioration if left untreated. The average distance between the alveolar crest and the enamel–cementum border is 2 mm, according to most research. In the present investigation, the gap between the alveolar crest and the enamel–cementum boundary was acknowledged as greater than 2 mm and classified according to the kind of destruction pattern.

**Figure 1:**
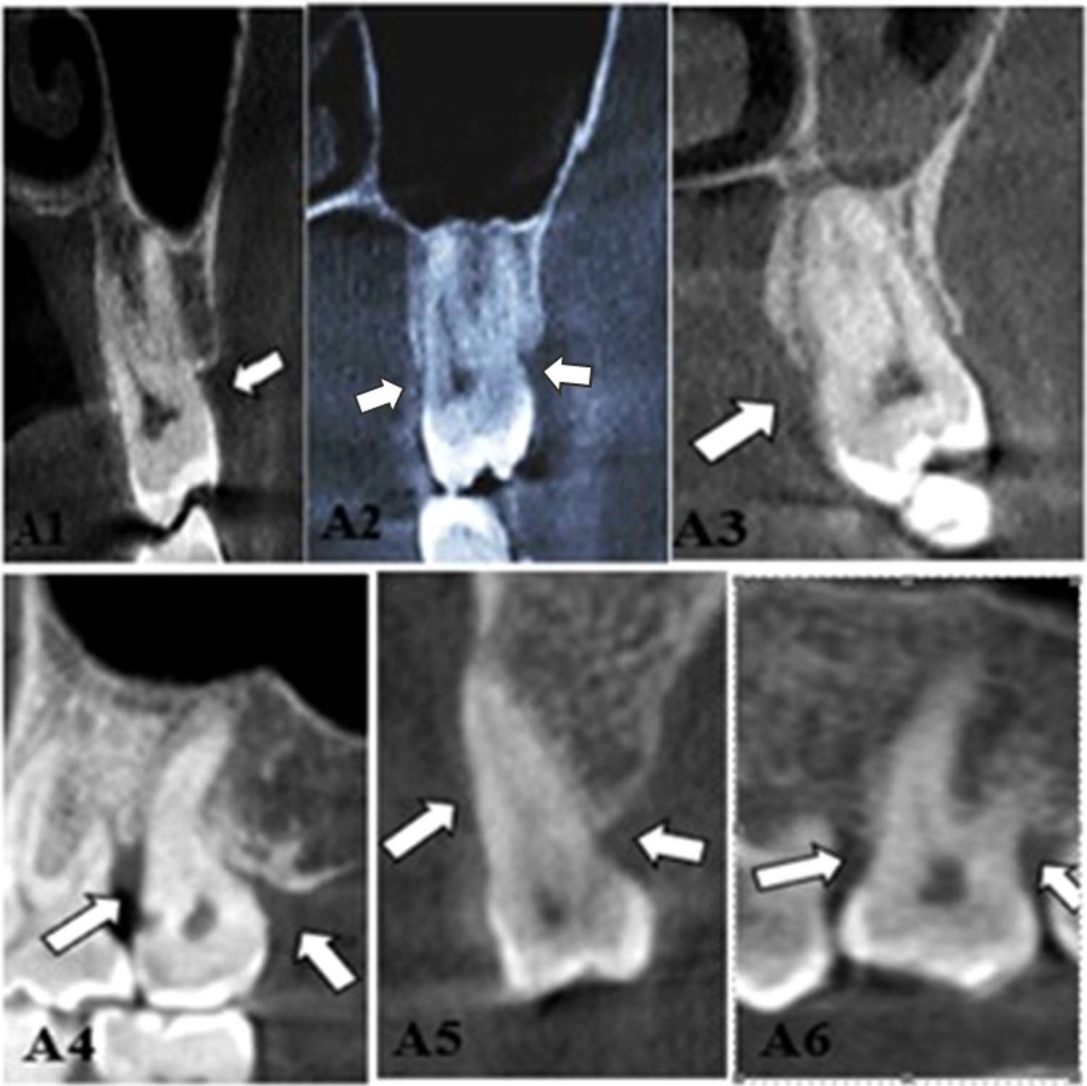
Destruction with a horizontal wall in the buccal region in the coronal section (A1). Horizontal two‐walled destruction in both buccal and palatinal section in the coronal section (A2). Three‐walled horizontal destruction in palatinal, mesial, and distal regions in coronal and sagittal sections, respectively (A3, A4). Combined horizontal destruction in buccal, palatinal, mesial, and distal regions in coronal and sagittal sections, respectively (A5, A6) (marked with a white arrow).

**Figure 2:**
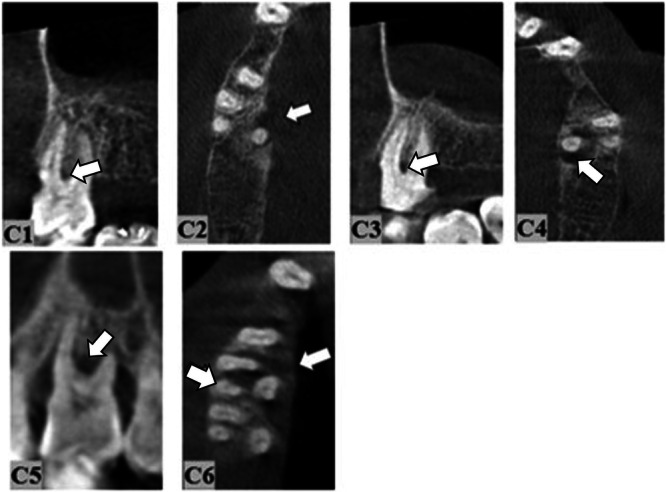
Grade 1 furcation involvement in the sagittal section (C1). Grade 1 furcation involvement in the axial section (C2). Grade 2 furcation involvement in the sagittal section (C3). Grade 2 furcation involvement in the axial section (C4). Grade 3 furcation involvement in the sagittal section (C5). Grade 3 furcation involvement in the axial section (C6) (marked with a white arrow).

**Figure 3:**
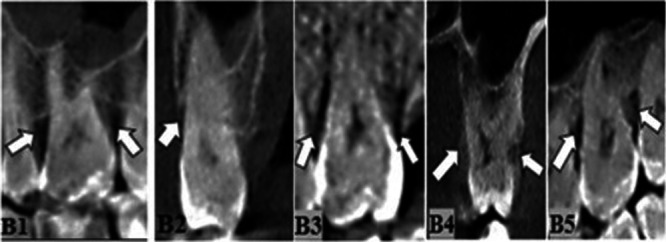
Vertical two‐walled destruction in mesial and distal regions in the sagittal section (B1). Vertical three‐walled destruction in buccal, mesial, and distal regions in coronal and sagittal sections, respectively (B2, B3). Vertical combined destruction in buccal, palatinal, mesial, and distal regions in coronal and sagittal sections, respectively (B4, B5) (marked with a white arrow).

Horizontal and vertical destructions, craters, furcation involvement, dehiscence, fenestration, and mixed periodontal–endodontic lesions are the seven major types of periodontal bone loss (CPEL). Vertical destructions in our study were categorized as one‐walled, two‐walled, three‐walled, or combined defects on the basis of the number of osseous walls, according to Goldman and Cohen (1958) (Figures 1, 2, 3).

The Irving–Glickman classification was used for the classification of furcation involvement. Furcation involvements were graded as Grades 1, 2, and 3 according to Glickman (1972):

Grade 1: Involves the loss of horizontal alveolar bone that reaches the furcation groove of the base of the pocket without an intraosseous defect.
Grade 2: Involves the loss of alveolar bone in both horizontal and vertical directions with a pronounced pocket depth that has not advanced enough to merge with the lesion coming from the other region into the furcation area.
Grade 3: Involves the loss of alveolar bone in the area between the roots that allows the periodontal probe to pass from one face to the other.

### 2.2 Statistical Analysis

IBM SPSS Statistics 22 (IBM SPSS, Turkey) program was used for statistical analysis. While evaluating the study data, the suitability of the parameters to a normal distribution was evaluated using the Kolmogorov–Smirnov test. After use of descriptive statistical methods (mean, standard deviation, and frequency), the Kruskal–Wallis test was used for the comparison of the parameters between the groups for quantitative data. The Mann–Whitney *U*‐test was used for comparisons of parameters between the two groups. For comparison of qualitative data, the chi‐squared test, Fisher's exact chi‐squared test, continuity (Yates) correction, and the Fisher–Freeman–Halton test were used. Significance was considered at the *p* < 0.05 level.

## 3 Results

The present study examined 1444 teeth of 361 patients ranging in age from 20 to 63 years, with an average age of 34.11 ± 9.71 years. One hundred and eighty (49.9%) patients were male and 181 patients (50.1%) were female.

Horizontal defects were one‐walled in 11.5%, two‐walled in 15.9%, three‐walled in 12%, and combined in 35% of the cases and the affected alveolar bone was mesial in 60.2%, distal in 60.7%, buccal in 51.4%, and palatinal in 46.5% of cases. Two‐walled vertical defects were 0.5% two‐walled, 0.6% three‐walled, and 0.3% combined, whereas 1.2% were located mesially, 1% distally, 0.9% buccally, and 0.6% palatinally. Crater was seen in 38.5%, Grade 1 in 3.8%, Grade 2 in 1.9%, and Grade 3 furcation involvement in 0.5% of the cases. Buccal dehiscence was observed in 0.6%, palatinal in 1.1%, buccal and palatinal dehiscence in 0.3%, and CPEL in 4.3% of the cases (Table 1).

**Table 1:** Distributions of bone resorption parameters.

Bone resorption type and location	*n*	%
Horizontal		
One‐walled	166	11.5
Two‐walled	229	15.9
Three‐walled	174	12
Combined	506	35
Mesial	869	60.2
Distal	877	60.7
Buccal	742	51.4
Palatinal	672	46.5
Vertical		
One‐walled	0	0
Two‐walled	7	0.5
Three‐walled	8	0.6
Combined	4	0.3
Mesial	17	1.2
Distal	15	1
Buccal	13	0.9
Palatinal	8	0.6
Crater		
None	888	61.5
Visible	556	38.5
Furcation involvement		
None	1355	93.8
Grade 1	55	3.8
Grade 2	27	1.9
Grade 3	7	0.5
Dehiscence		
None	1414	97.9
Buccal	9	0.6
Palatinal	16	1.1
Buccal + palatinal	5	0.3
CPEL		
None	1382	95.7
Visible	62	4.3

The rate of one‐walled horizontal destruction was significantly higher in the left molar teeth than in the right molar teeth. The rate of three‐walled horizontal destruction in the right molar teeth was found to be statistically higher than the rate in the left molar teeth (Figure 1). The rate of Grade 1 and Grade 3 furcation involvement in the left molar teeth was considerably higher than the rate in the right molar teeth. The rate of Grade 2 furcation involvement in the right molar teeth was statistically higher than the rate in the left molar teeth (Table 2 and Figure 2).

**Table 2:** Evaluation of bone resorption parameters according to right and left molar teeth.

Bone resorption type and location	Right molar teeth, *n* (%)	Left molar teeth, *n* (%)	*p*
Horizontal			
One‐walled	66 (9.1%)	100 (13.9%)	0.005^a^ ^,^ *
Two‐walled	124 (17.2%)	105 (14.5%)	0.171^a^
Three‐walled	104 (14.4%)	70 (9.7%)	0.006^a^ ^,^ *
Combined	247 (34.2%)	259 (35.9%)	0.508^a^
Mesial	443 (61.4%)	426 (59%)	0.361^a^
Distal	449 (62.2%)	428 (59.3%)	0.258^a^
Buccal	378 (52.4%)	364 (50.4%)	0.461^a^
Palatinal	336 (46.5%)	336 (46.5%)	1.000^a^
Vertical			
Two‐walled	2 (0.3%)	5 (0.7%)	0.452^b^
Three‐walled	5 (0.7%)	3 (0.4%)	0.726^b^
Combined	2 (0.3%)	2 (0.3%)	1.000^b^
Mesial	8 (1.1%)	9 (1.2%)	1.000^c^
Distal	7 (1%)	8 (1.1%)	1.000^c^
Buccal	8 (1.1%)	5 (0.7%)	0.577^c^
Palatinal	4 (0.6%)	4 (0.6%)	1.000^b^
Crater			
Visible	280 (38.8%)	276 (38.2%)	0.829^a^
None	442 (61.2%)	446 (61.8%)	
Furcation involvement			
None	678 (93.9%)	677 (93.8%)	0.000^d^ ^,^ *
Grade 1	23 (3.2%)	32 (4.4%)	
Grade 2	21 (2.9%)	6 (0.8%)	
Grade 3	0 (0%)	7 (1%)	
Dehiscence			
None	705 (97.6%)	709 (98.2%)	0.077^d^
Buccal	8 (1.1%)	1 (0.1%)	
Palatinal	6 (0.8%)	10 (1.4%)	
Buccal + palatinal	3 (0.4%)	2 (0.3%)	
CPEL			
Visible	36 (5%)	26 (3.6%)	0.194^a^
None	686 (95%)	696 (96.4%)	

^a^ Chi‐squared test.

^b^ Fisher's exact test.

^c^ Continuity (Yates) correction.

^d^ Fisher–Freeman–Halton test.

* *p* < 0.05.

Males were shown to have statistically higher frequencies of horizontal defects when defects were combined or distally and palatinally located. Vertical defects with three walls and vertical defects located either distally or palatinally were found to be higher in males than females. Males had significantly more visible craters and higher Grade 1, 2, and 3 furcation involvement frequency compared to females (Table 3 and Figure 3).

**Table 3:** Comparison of bone resorption parameters in right and left molar teeth by gender.

Bone resorption type and location	Male, *n* (%)	Female, *n* (%)	*p*
Horizontal			
One‐walled	71 (9.9%)	95 (13.1%)	0.052^a^
Two‐walled	110 (15.3%)	119 (16.4%)	0.547^a^
Three‐walled	91 (12.6%)	83 (11.5%)	0.493^a^
Combined	273 (37.9%)	233 (32.2%)	0.022^a^ ^,^ *
Mesial	450 (62.5%)	419 (57.9%)	0.073^a^
Distal	460 (63.9%)	417 (57.6%)	0.014^a^ ^,^ *
Buccal	380 (52.8%)	362 (50%)	0.291^a^
Palatinal	360 (50%)	312 (43.1%)	0.009^a^ ^,^ *
Vertical			
Two‐walled	2 (0.3%)	5 (0.7%)	0.452^b^
Three‐walled	7 (1%)	1 (0.1%)	0.038^b^ ^,^ *
Combined	4 (0.6%)	0 (0%)	0.062^b^
Mesial	12 (1.7%)	5 (0.7%)	0.140^c^
Distal	12 (1.7%)	3 (0.4%)	0.037^c^ ^,^ *
Buccal	9 (1.3%)	4 (0.6%)	0.261^c^
Palatinal	8 (1.1%)	0 (0%)	0.004^b^ ^,^ *
Crater			
Visible	461 (64%)	427 (59%)	0.049^a^ ^,^ *
None	259 (36%)	297 (41%)	
Furcation involvement			
None	656 (91.1%)	699 (96.5%)	0.000^d^ ^,^ *
Grade 1	39 (5.4%)	16 (2.2%)	
Grade 2	20 (2.8%)	7 (1%)	
Grade 3	5 (0.7%)	2 (0.3%)	
Dehiscence			
None	703 (97.6%)	711 (98.2%)	0.064^d^
Buccal	6 (0.8%)	3 (0.4%)	
Palatinal	6 (0.8%)	10 (1.4%)	
Buccal + palatinal	5 (0.7%)	0 (0%)	
CPEL			
Visible	684 (95%)	698 (96.4%)	0.187^a^
None	36 (5%)	26 (3.6%)	

^a^ Chi‐squared test.

^b^ Fisher's exact test.

^c^ Continuity (Yates) correction.

^d^ Fisher–Freeman–Halton test.

* *p* < 0.05.

Females had a statistically higher rate of one‐walled horizontal damage in the right molar teeth than males. Males had statistically higher rates of combined horizontal damage, horizontal destruction in the palatinal region, crater formation, and Grade 1 and Grade 2 furcation involvement than females (Table 4).

**Table 4:** Evaluation of bone resorption parameters of right and left molar teeth by gender.

Right molar teeth	Resorption type and location	Male, *n* (%)	Female, *n* (%)	*p*	Left molar teeth	Resorption type and location	Male, *n* (%)	Female, *n* (%)	*p*
Horizontal	One‐walled	21 (5.8%)	45 (12.4%)	0.002^a^ ^,^ *	Horizontal	One‐walled	50 (13.9%)	50 (13.8%)	0.976^a^
	Two‐walled	60 (16.7%)	64 (17.7%)	0.718^a^		Two‐walled	50 (13.9%)	55 (15.2%)	0.619^a^
	Three‐walled	51 (14.2%)	53 (14.6%)	0.856^a^		Three‐walled	40 (11.1%)	30 (8.3%)	0.200^a^
	Combined	138 (38.3%)	109 (30.1%)	0.020^a^ ^,^ *		Combined	135 (37.5%)	124 (34.3%)	0.363^a^
	Mesial	228 (63.3%)	215 (59.4%)	0.277^a^		Mesial	222 (61.7%)	204 (56.4%)	0.147^a^
	Distal	235 (65.3%)	214 (59.1%)	0.088^a^		Distal	225 (62.5%)	203 (56.1%)	0.079^a^
	Buccal	195 (54.2%)	183 (50.6%)	0.331^a^		Buccal	185 (51.4%)	179 (49.4%)	0.602^a^
	Palatinal	184 (51.1%)	152 (42%)	0.014^a^ ^,^ *		Palatinal	176 (48.9%)	160 (44.2%)	0.207^a^
Vertical	Two‐walled	1 (0.3%)	1 (0.3%)	1.000^b^	Vertical	Two‐walled	1 (0.3%)	4 (1.1%)	0.373^b^
	Three‐walled	4 (1.1%)	1 (0.3%)	0.216^b^		Three‐walled	3 (0.8%)	0 (0%)	0.123^b^
	Combined	2 (0.6%)	0 (0%)	0.248^b^		Combine	2 (0.6%)	0 (0%)	0.248^b^
	Mesial	6 (1.7%)	2 (0.6%)	0.176^b^		Mesial	6 (1.7%)	3 (0.8%)	0.340^b^
	Distal	6 (1.7%)	1 (0.3%)	0.068^b^		Distal	6 (1.7%)	2 (0.6%)	0.176^b^
	Buccal	6 (1.7%)	2 (0.6%)	0.176^b^		Buccal	3 (0.8%)	2 (0.6%)	0.686^b^
	Palatinal	4 (1.1%)	0 (0%)	0.061^b^		Palatinal	4 (1.1%)	0 (0%)	0.061^b^
Crater	Visible	234 (65%)	208 (57.5%)	0.038^a^ ^,^ *	Crater	Visible	227 (63.1%)	219 (60.5%)	0.479^a^
	None	126 (35%)	154 (42.5%)			None	133 (36.9%)	143 (39.5%)	
Furcation involvement	None	327 (90.8%)	351 (97%)	0.002^a^ ^,^ *	Furcation involvement	None	329 (91.4%)	348 (96.1%)	0.058^d^
	Grade 1	18 (5%)	5 (1.4%)			Grade 1	21 (5.8%)	11 (3%)	
	Grade 2	15 (4.2%)	6 (1.7%)			Grade 2	5 (1.4%)	1 (0.3%)	
	Grade 3	0	0			Grade 3	5 (1.4%)	2 (0.6%)	
Dehiscence	None	350 (97.2%)	355 (98.1%)	0.239^d^	Dehiscence	None	353 (98.1%)	356 (98.3%)	0.351^d^
	Buccal	5 (1.4%)	3 (0.8%)			Buccal	1 (0.3%)	0 (0%)	
	Palatinal	2 (0.6%)	4 (1.1%)			Palatinal	4 (1.1%)	6 (1.7%)	
	Buccal + palatinal	3 (0.8%)	0 (0%)			Buccal + palatinal	2 (0.6%)	0 (0%)	
CPEL	Visible	338 (93.9%)	348 (96.1%)	0.225^c^	CPEL	Visible	346 (96.1%)	350 (96.7%)	0.830^c^
	None	22 (6.1%)	14 (3.9%)			None	14 (3.9%)	12 (3.3%)	

^a^ Chi‐squared test.

^b^ Fisher's exact test.

^c^ Continuity (Yates) correction.

^d^ Fisher–Freeman–Halton test.

* *p* < 0.05.

The average age of patients with one‐walled horizontal destruction was substantially lower than the average age of patients without one‐walled destruction in the right and left molars. The mean age of patients with horizontal two‐walled, three‐walled, combination, mesial, distal, buccal, and palatinal destruction was shown to be significantly higher than the average age of patients without these destructions. The average age of patients with right and left molar crater formation was significantly lower than the average age of patients without crater formation. The average age of patients with Grade 1 furcation participation was significantly higher than the average age of patients with Grade 3 furcation involvement. Patients with Grade 2 furcation were considerably older in terms of the mean age than patients without Grade 2 furcation involvement. The average age of patients with buccal surface dehiscence was much lower than the age of patients with buccal + palatinal surface dehiscence. The average age of CPEL patients was statistically considerably older than the average age of non–CPEL patients (Table 5).

**Table 5:** Evaluation of bone resorption parameters in right and left molar teeth according to age.

	Age	
Resorption type and location	Mean ± SD (median)	*p*
One‐walled horizontal		
None	34.9 ± 9.9 (34)	0.000*
Visible	28 ± 5.4 (27)	
Two‐walled horizontal		
None	33.9 ± 10.1 (31)	0.006*
Visible	35 ± 7.1 (36)	
Three‐walled horizontal		
None	33.6 ± 9.9 (31)	0.000*
Visible	38.1 ± 6.4 (38.5)	
Combined horizontal		
None	30.7 ± 7.7 (29)	0.000*
Visible	40.5 ± 9.9 (42)	
Mesial horizontal		
None	28 ± 6.9 (26)	0.000*
Visible	38.2 ± 9.2 (38)	
Distal horizontal		
None	27.7 ± 6.6 (26)	0.000*
Visible	38.2 ± 9.2 (39)	
Buccal horizontal		
None	28.7 ± 6.6 (27)	0.000*
Visible	39.2 ± 9.4 (40)	
Palatinal horizontal		
None	29.6 ± 7.1 (28)	0.000*
Visible	39.2 ± 9.8 (40)	
Two‐walled vertical		
None	34.1 ± 9.7 (33)	0.440
Visible	30.6 ± 4.5 (31)	
Three‐walled vertical		
None	34.1 ± 9.7 (33)	0.316
Visible	36.3 ± 5.5 (34.5)	
Combined vertical		
None	34.1 ± 9.7 (33)	0.889
Visible	32 ± 2.3 (32)	
Mesial vertical		
None	34.1 ± 9.7 (33)	0.729
Visible	33.7 ± 5 (34)	
Distal vertical		
None	34.1 ± 9.7 (33)	0.524
Visible	34.3 ± 4.6 (34)	
Buccal vertical		
None	34.1 ± 9.7 (33)	0.973
Visible	33.1 ± 5.7 (34)	
Palatinal vertical		
None	34.1 ± 9.7 (33)	0.842
Visible	33.4 ± 3.7 (34)	
Crater		
None	37.9 ± 9.5 (38)	0.000*
Visible	28 ± 6.4 (27)	
Furcation involvement		
None	33.4 ± 9.3 (32)	0.000*
Grade 1	46.2 ± 7.9 (47)	
Grade 2	44 ± 9.5 (45)	
Grade 3	37.7 ± 12.9 (30)	
Dehiscence		
None	33.9 ± 9.7 (32)	0.000*
Buccal	39.9 ± 7.2 (41)	
Palatinal	44.5 ± 5.8 (47)	
Buccal + palatinal	47.4 ± 2.5 (48)	
CPEL		
None	33.9 ± 9.7 (32)	0.000*
Visible	39.2 ± 9 (39)	

*Note:* Kruskal–Wallis test was used for furcation involvement and dehiscence; the Mann–Whitney *U*‐test was used for other destruction parameters.

* *p* < 0.05.

The incidence of one‐ and two‐walled horizontal destruction in right and left molar teeth with no furcation destruction was statistically greater than those in patients with Grade 1, Grade 2, and Grade 3 furcation involvement. The rate of horizontal damage in the combined mesial, distal, buccal, and palatinal areas was considerably lower in patients with no furcation participation than in patients with Grade 1, Grade 2, and Grade 3 furcation involvement. The rate of horizontal damage in the buccal region was much lower in patients with Grade 3 furcation involvement than in those with Grade 1 furcation involvement. The rate of three‐walled, combined, mesial, distal, buccal, and palatinal vertical damage was considerably greater in patients with Grade 3 furcation than in patients with no furcation involvement or Grade 1 furcation involvement. Patients with Grade 1, Grade 2, and Grade 3 furcation involvement had a considerably greater incidence of crater than patients with no furcation involvement.

The rate of no dehiscence in cases with no furcation involvement was significantly higher than the rate of no dehiscence in patients with Grade 1, Grade 2, and Grade 3 furcation involvement. The rate of no dehiscence in patients with Grade 1 furcation involvement was significantly higher than the rate of no dehiscence in patients with Grade 2 furcation involvement. The frequency of CPEL was much lower in patients with Grade 2 furcation involvement than in those with Grade 1 and Grade 3 furcation involvement (Table 6).

**Table 6:** Comparison of furcation and other bone resorption parameters in the right and left molar teeth.

	Furcation involvement				
Bone resorption type and location	None, *n* (%)	Grade 1, *n* (%)	Grade 2, *n* (%)	Grade 3, *n* (%)	*p*
Horizontal					
One‐walled	166 (12.3%)	0 (0%)	0 (0%)	0 (0%)	0.002*
Two‐walled	229 (16.9%)	0 (0%)	0 (0%)	0 (0%)	0.000*
Three‐walled	167 (12.3%)	6 (10.9%)	1 (3.7%)	0 (0%)	0.576
Combined	428 (31.6%)	49 (89.1%)	24 (88.9%)	5 (71.4%)	0.000*
Mesial	786 (58%)	53 (96.4%)	25 (92.6%)	5 (71.4%)	0.000*
Distal	793 (58.5%)	54 (98.2%)	25 (92.6%)	5 (71.4%)	0.000*
Buccal	657 (48.5%)	55 (100%)	25 (92.6%)	5 (71.4%)	0.000*
Palatinal	591 (43.6%)	52 (94.5%)	24 (88.9%)	5 (71.4%)	0.000*
Vertical					
Two‐walled	7 (0.5%)	0 (0%)	0 (0%)	0 (0%)	1.000
Three‐walled	6 (0.4%)	0 0%)	1 (3.7%)	1 (14.3%)	0.014*
Combined	2 (0.1%)	0 (0%)	1 (3.7%)	1 (14.3%)	0.002*
Mesial	13 (1%)	0 (0%)	2 (7.4%)	2 (28.6%)	0.000*
Distal	11 (0.8%)	0 (0%)	2 (7.4%)	2 (28.6%)	0.000*
Buccal	10 (0.7%)	0 (0%)	2 (7.4%)	1 (14.3%)	0.004*
Palatinal	5 (0.4%)	0 (0%)	1 (3.7%)	2 (28.6%)	0.000*
Crater					
Visible	555 (41%)	0 (0%)	1 (3.7%)	0 (0%)	0.000*
Dehiscence					
None	1351 (99.7%)	44 (80%)	14 (51.9%)	5 (71.4%)	0.000*
Buccal	2 (0.1%)	2 (3.6%)	5 (18.5%)	0 (0%)	
Palatinal	1 (0.1%)	9 (16.4%)	4 (14.8%)	2 (28.6%)	
Buccal + palatinal	1 (0.1%)	0 (0%)	4 (14.8%)	0 (0%)	
Visible	54 (4%)	7 (12.7%)	0 (0%)	1 (14.3%)	0.011*

* *p* < 0.05, using Fisher–Freeman–Halton test.

## 4 Discussion

Periodontitis is an inflammatory condition mediated by the host and can result in a wide range of problems. Because of their complicated root morphology, characterized by the concavity of the root surface and furcation, maxillary molars are particularly susceptible to periodontal infection (Al‐Shammari, Kazor, and Wang 2001). Additionally, maxillary molar teeth are lost more frequently due to periodontitis than teeth in other regions, and the maxillary molar area has been identified as the most difficult to treat (Sánchez‐Pérez and Moya‐Villaescusa 2009; Laurell, Romao, and Hugoson 2003).

In this study, radiographic features of the alveolar bone pattern and associated periodontal defects in 1444 maxillary molars were comprehensively evaluated with CBCT. According to the findings, both horizontal defects and three‐walled vertical defects were located distally and palatinally and were higher in males than females, whereas age was not found to be a determinant factor for any of the mentioned defects.

CBCT was used in this study due to ample data provided in the literature of its superiority compared to periapical radiographs. CBCT is a more accurate approach in assessing the buccal–lingual and palatinal surfaces and imaging the morphology of the destruction compared to intraoral radiographs (de Faria Vasconcelos et al. 2012). It also provides a more thorough radiographic approach for diagnosing periodontal abnormalities such as intraosseous defects, dehiscence formations, fenestrations, and furcation defects (Braun et al. 2014). Moreover, CBCT has been reported to have a stronger association with surgical measures than intraoral radiographs and was a highly effective radiographic tool for monitoring the outcomes of regenerative surgical therapy (Grimard et al. 2009). The accuracy of CBCT in measuring horizontal periodontal bone defects was also studied by comparing CBCT measurements to clinical measurements. No significant difference was reported between both measurements, indicating that CBCT could accurately assess the horizontal periodontal bone abnormalities (Feijo et al. 2012).

In the present investigation, CBCT was used to assess horizontal destruction in single‐, double‐, triple‐walled, and combined horizontal defects on four different surfaces (mesial, distal, buccal, and palatal). Except for single‐walled destructions, it has been found that the frequency of horizontal destruction increases with age.

On the distal (Nielsen, Glavind, and Karhing 1980) and mesial (Papapanou and Tonetti 2000) surfaces, radiographically identified vertical defects are a common occurrence, and their frequency increases with age (Wouters et al. 1989). However, according to Larato, the mesial surfaces of the upper and lower molars were more frequently affected by three‐walled abnormalities (Larato 1970). In the study of Özcan and Şekerci, vertical deficiencies were more common on the mesial and distal surfaces than on the buccal and palatal surfaces (single‐walled, two‐walled, three‐walled, and combination defects). Additionally, it was discovered that three‐walled deficiencies were more prevalent on mesial surfaces than on other surfaces and, similar to other studies, the prevalence of vertical defects was disproportionately high in those older than 40 years of ag (Özcan and Şekerci 2017). However, there was no difference in the findings of the present study regarding the prevalence of age‐related vertical defects on the buccal, palatal, mesial, and distal surfaces (single‐walled, two‐walled, and combined defects). Moreover, the prevalence of the location of the vertical defects differed compared to previous studies (Özcan and Şekerci 2017; Vandenberghe, Jacobs, and Yang 2008).

In this study, the relationship between gender and horizontal and vertical destruction of maxillary molars was also evaluated. Although Özcan and Şekerci (2017) did not find a relationship between gender and the pattern of alveolar bone loss in the maxillary molar region, in the present study, the frequency of horizontal combined, distal, and palatal destruction and vertical three‐walled, distal, and palatal destruction was found to be higher in males.

The difference in the impact of age and gender as well as the location of defects might be attributed to the difference in study groups, study design, and geography.

One limitation of this study is its retrospective nature and, therefore, the inability to draw any conclusion regarding the frequency. Due to already presented advantages of CBCT over periapical radiographs, a comparison with radiographs was not carried out in this study. Future investigations should compare CBCT with periapical radiographs. Another limitation of the study was the exclusion of mandibular molars and the initial periodontal status of the subjects. Therefore, the data should be interpreted with caution.

## 5 Conclusions

According to the CBCT evaluations, alveolar bone loss pattern in maxilla was affected by age and gender. Due to multifactorial features of the alveolar bone loss, more studies should be performed with reliable imaging techniques such as CBCT to better understand the factors that affect alveolar bone loss.

## Author Contributions

All authors confirm that they equally contributed to the study conception, design, data collection, analysis, interpretation of results, and manuscript preparation.

## Conflicts of Interest

The authors declare no conflicts of interest.

## Data Availability

The data sets used and/or analyzed during the current study are available from the corresponding author upon reasonable request.
